# The ^18^F-PSMA-1007 PET/CT performance on metastasis status and therapy assessment in oligo-metastasis prostate cancer

**DOI:** 10.3389/fonc.2022.935979

**Published:** 2022-08-26

**Authors:** Zhuonan Wang, Anqi Zheng, Yunxuan Li, Jungang Gao, Weixuan Dong, Yan Li, Xiaoyi Duan

**Affiliations:** PET/CT Center, The First Affiliated Hospital of Xi’an Jiaotong University, Xi’an, China

**Keywords:** oligo-metastatic, prostate cancer, SUVmax, androgen deprivation therapy, ^18^F-PSMA-1007 PET/CT

## Abstract

**Objective:**

The prostate-specific membrane antigen (PSMA) PET/CT is potentially identifying patients with oligo-metastasis who would be deemed to only have localized disease in the traditional approaches. However, the best selected oligo-metastasis prostate cancer (PCa) patients most likely to benefit from system androgen deprivation therapy (ADT) are still unknown. The aim of this study was to explore the potential ^18^F-PSMA-1007 PET/CT parameters and clinicopathologic characteristics for oligo-metastasis PCa discrimination and follow-up evaluation.

**Materials and methods:**

A total of 180 retrospective patients with different metastasis burdens (PCa of none-metastases, oligo-metastases, and poly-metastases), different metastasis status (untreated and recurrent oligo-metastases), and follow-up ADT were included respectively. A one-way analysis of variance was used to evaluate whether PET/CT parameters and clinicopathologic characteristics were different and univariate/multivariate logistic regression models were applied to assess independent predictors in the metastasis burdens group (89/180). Selected predictors were further compared between different metastasis statuses to test the diagnostic accuracy (69/180). The predictor efficiency was evaluated by the ROC and the cut-off value was used to test the ADT response-to-treatment with a longitudinal cohort (22/180) from untreated baseline to 3-15 months.

**Results:**

The significant group differences were observed on SUVmax (P = 0.012), International Society of Urologic Pathologists (ISUP, P<0.001) and Gleason Score (P<0.001). Poly-Metastases patients had higher SUVmax, ISUP and Gleason Score compared to Non-Metastases and Oligo-Metastases patients, respectively (P<0.05, all), and no difference between Non-Metastases and Oligo-Metastases. The SUVmax, ISUP and Gleason Score were independent predictors for metastasis burdens discrimination. The untreated and recurrent oligo-metastases lesions SUVmax were also different (P = 0.036). The AUC of ROC for oligo-metastasis prediction was 0.658 (P = 0.039) when the primary prostatic carcinoma focus SUVmax was higher than 28.22, ADT response-to-treatment patients (5/5 in 22) were all progress in a follow-up test.

**Conclusion:**

The SUVmax can discriminate PCa metastasis degree and oligo-metastasis status. The ADT-treated oligo-metastasis patient may still have disease progression when the primary prostatic carcinoma focus SUVmax is greater than 28.22.

## Introduction

The oligo-metastasis state has been proposed as an intermediate stage of cancer spread between local disease and widespread metastasis (1). Hellman and Weichselbaum first proposed a clinically significant state of oligo-metastasis in 1995 (2) and Singh and colleges were the first to use the term oligo-metastasis disease in the setting of prostate cancer (PCa) (3). The clinical diagnosis made on the basis of up to five extra-pelvic lesions is reasonable to the definition (1). Due to the limited number of metastases involved and the early derivation of monoclonal amplification, which may spread to other sites over time, the goal of clinical staging in oligo-metastasis PCa is to determine the burden of disease and predict the prognosis *via* pretreatment clinical parameters to direct the patient for the most benefit in the decision-making strategy of treatment (4, 5).

With the advent of newly imaging technology prostate-specific membrane antigen (PSMA) PET/CT has significantly increased the detection rate of extra-pelvic metastases of PCa, providing more clinical reference for the treatment of oligo-metastasis disease (6–8). However, a proportion of micro-metastatic disease remains occult using conventional imaging and ^68^Gallium PMSA PET/CT, which poses ongoing challenges to maximizing the benefit of oligo-metastasis treatment options (9). Recent studies on the mechanism of tumor metastasis have shown that in addition to early metastasis from the primary tumor, the metastasis itself may also become a source of further metastasis (10). With the heterogeneity of the source of metastasis, there is provider and patient bias toward certain treatments. The options include androgen deprivation therapy (ADT) alone, focused radical prostatectomy alone with imaging and prostate-specific antigen (PSA) dynamic monitoring, or both (11). Although studies show prior radical prostatectomy has been associated with improved overall survival for PCa with pelvic lymph node metastatic, ADT therapy for controlling the disease progress and oligo-metastasis patients selection remains unknown (12, 13).

The main aim of the present study, therefore, was to investigate the potential PET/CT parameters and clinicopathologic characteristics for oligo-metastasis patients discrimination and follow-up evaluation. This could provide the basis for monitoring the progress of oligo-metastasis disease on ^18^F-PSMA-1007 PET/CT, and benefit from ADT treatment in the primary stage.

## Materials and methods

### Patients

**Figure 1:**
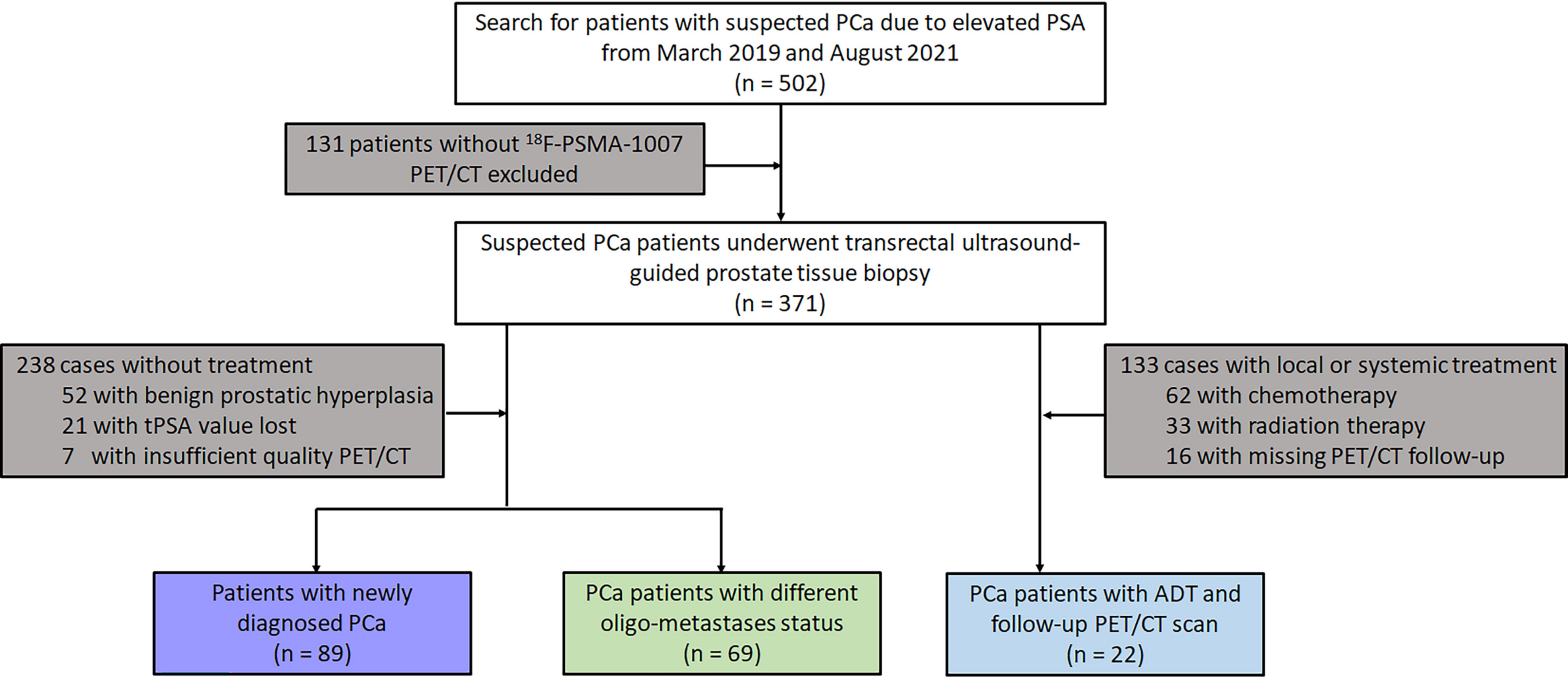
Flowchart of the prostate cancer patient’s cohort.

One hundred and eighty PCa patients confirmed by transrectal ultrasound-guided prostate tissue biopsies from March 2019 and August 2021 were retrospectively identified and enrolled. All patients were divided into three subgroups according to whether they were treated or not and oligo- metastasis statuses follow: First, 89 newly diagnosed untreated PCa who were referred for ^18^F-PSMA-1007 PET/CT for primary staging were analysed. Secondly, 69 patients were also included in the analysis for testing the different oligo-metastasis statuses (metastases before treatment or after radical prostatectomy). Finally, 22 follow-up patient intervals ranging between 3 and 15 months who received ADT treatment were assessed by ^18^F-PSMA-1007 PET/CT according to Practical PERCIST 1.0 and Consensus statements on PSMA PET/CT response assessment for disease progression assessment (13–15). Diagnosis of PCa proven through histological examination served as the reference for the PET imaging analyses (16). If the patient subsequently underwent radical prostatectomy, we compared the pathological findings with TRUS and selected a higher-grade Gleason score as the criterion. Patients were excluded from analysis if they 1) lacked histological examination proven diagnosis or PSA value, 2) had incomplete imaging data, 3) underwent systemic or local treatment after radical prostatectomy. The flowchart of patient enrollment is provided in **Figure 1**.

### ^18^F-PSMA-1007 and image acquisition

All ^18^F-PSMA-1007 PET/CT data were acquired on a PET/CT scanner (Gemini 64TF, Philips, Netherlands) at a single location. Radiolabeling was performed using a fully automated radiopharmaceutical synthesis device based on a modular concept (MINItrace, GE Healthcare, USA). Over 99% radiochemical purification yield ^18^F-PSMA-1007 was obtained and examined by both radiothin layer chromatography and high-performance liquid chromatography analysis. Patients received intravenous injection of ^18^F-PSMA-1007 PET/CT (3.7 MBq/kg body weight), and completed PET and CT scan 90 minutes after the injection. Low-dose CT scans from the head to the proximal thighs (pitch 0.8 mm, 60 mA, 140 kV [peak], tube single turn rotation time 1.0 s and 5-mm slice thickness) for PET attenuation were acquired (pitch 0.8 mm, automatic mA, 140 kV [peak] and 512 × 512 matrix). Whole-body PET scans were performed in three-dimensional mode (emission time: 90 s per bed position, scanned at a total of 7-10 beds) as in our previous study (17).

### Imaging analysis

Two experienced board-certified nuclear medicine specialists jointly interpreted all ^18^F-PSMA-1007 PET/CT scans, using Fusion Viewer software in the Extended Brilliance Workstation (EBW, Philips, Netherlands), and performed a comprehensive analysis of available clinical data. Consensuses were achieved through discussion when conclusions between the two specialists were discordant. The Maximum and Mean Standardized Uptake Value (SUVmax and SUVmean) for ^18^F-PSMA-1007 PET/CT of the PCa was calculated automatically with a manually adapted isocontour threshold centered on lesions with focally increased uptake corresponding to the tumor site verified by TRUS biopsy (18). The SUVmax was also calculated for metastases lesions.

### Definition of metastatic lesions at ^18^F-PSMA-1007 PET/CT

The positive lesion was defined by an uptake higher than the local background and not associated with physiologic uptake per the guideline of the Society of Nuclear Medicine and Molecular Imaging and the European Association of Nuclear Medicine (19, 20). The oligo-metastatic positive lesions were defined as the presence of (a) consistent with PCa lesions tracer accumulation in the extra-pelvic lymph or in the bone and (b) a maximum of five lesions in the extra-pelvic lymph nodes or in the bone (21, 22). The positive metastasis lesions were also compositely validated by other imaging approaches, disease management and PSA measurements.

### Statistical analysis

Descriptive statistics were used to display patient data as mean, standard deviation range or percentages, where applicable. The PET/CT parameters and clinicopathologic characteristics were compared across sub-groups for newly diagnosed patients (PCa of none-metastases, oligo-metastases and poly-metastases) using analysis of variance (ANOVA). Logistic regression was used to identify independent predictors for metastases results. The independent two-sample t-Test and Mann–Whitney U test were used to compare different oligo-metastases status based on data normality and the best threshold of clinicopathologic and PET/CT parameters performance were assessed using receiver operating characteristic (ROC) curve analysis. A significance level of α = 0.05 (two-tailed) was applied. Statistical analyses were performed using IBM SPSS Statistics version 13.0 (IBM Corp., Armonk, NY, USA), GraphPad Prism software, version 8.4 (GraphPad Software, Inc., La Jolla, CA, USA) and MedCalc version 19.0 (MedCalc Software Ltd, Belgium).

## Results

**Figure 2:**
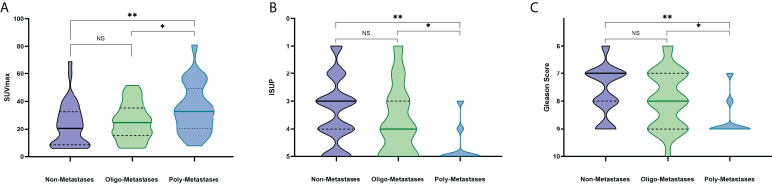
PET/CT parameters and clinicopathologic characteristics difference among the metastasis’s groups. NS: No statistical difference. *P < 0.05, **P < 0.01. **(A)** SUVmax, **(B)** ISUP, **(C)** Gleason Score.

Detailed information on 89 newly diagnosed patients’ characteristics is shown in **Table 1**. All patients presented with a median PSA value of 24.23 ng/ml and the Gleason Score among patients ranged from 6 to 10, International Society of Urological Pathology (ISUP) grade ranged from 1 to 5. The median SUVmax and SUVmean of all primary PCa lesions were 24.83 (range: 5.95-81.00) and 11.36 (range: 2.67-43.65), respectively. According to metastatic status, patients were divided into Non-Metastases, Oligo-Metastases and Poly-Metastases sub-groups. Significant between-group differences were observed on SUVmax (F = 4.636, P = 0.012), ISUP (F = 9.501, P<0.001) and Gleason Score (F = 9.592, P<0.001), but not in the SUVmean (F = 2.245, P = 0.112) and PSA value (F = 2.948, P = 0.058). Post-hoc comparisons revealed significant differences in the Non-Metastases vs Poly-Metastases and Oligo-Metastases vs Poly-Metastases sub-groups for these three parameters (SUVmax, ISUP and Gleason Score, P < 0.05, all), and no statistically significant difference was found between the Non-Metastases and Oligo-Metastases groups (P>0.05, all) (**Figure 2**).

**Table 1:** Demographic and clinical characteristics of the different metastatic status of PCa participants.

Characteristic	Value (Overall *n = 89*)
**Age (years)**	
Mean ± SD (range)	72.87 ± 8.23 (54-91)
**PSA (ng/mL)** Mean ± SD (range)	51.60 ± 67.87 (1.17-398.73)
**Metastatic Status**	
Non-Metastases Oligo-Metastases Poly-Metastases	37 (41.57%) 27 (30.34%) 25 (28.09%)
**Gleason Score** 6 7 8 9 10	5 (5.6%) 27 (30.3%) 21 (23.6%) 35 (39.3%) 1 (1.1%)
**ISUP Grade**	
1	5 (5.6%)
2	8 (9.0%)
3	19 (21.3%)
4	21 (23.6%)
5	36 (40.4%)

PCa, prostate cancer; SD, standard deviation; PSA, prostate-specific antigen; ISUP, International Society of Urological Pathology; ISUP Grade 1, Gleason ≤ 6; ISUP Grade 2, Gleason = 3 + 4; ISUP Grade 3, Gleason = 4 + 3; ISUP Grade 4, Gleason = 8; ISUP Grade 5, Gleason>8.

To further evaluate the diagnostic strength of metastatic discrimination, SUVmax, ISUP Grade and Gleason Score were entered as independent in a logistics regression. The univariate analysis identified the SUVmax, ISUP Grade, Gleason Score and PSA value as potential predictive factors for metastasis status. In multivariable analysis, these three variables were also identified as significant independent predictors (**Table 2**).

**Table 2:** Logistic analyses of factors differentiate metastasis status.

	Univariate Analysis		Multivariable Analysis	
	OR (95% CI)	*P* value	OR (95% CI)	*P* value
SUVmax	2.642 (1.052-2.985)	<0.001	2.985 (1.126-7.676)	<0.001
ISUP Grade	3.519 (1.095-5.630)	<0.001	2.477 (0.784-7.828)	0.036
Gleason Score	3.604 (1.197-10.848)	<0.001	2.252 (0.702-7.225)	0.043

(Non-Metastases vs Oligo-Metastases vs Poly-Metastases).

**Figure 3:**
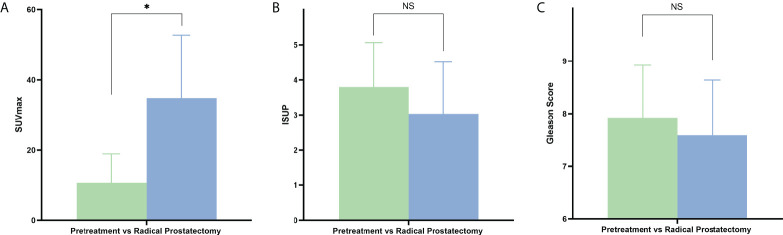
PET/CT parameters and clinicopathologic characteristics difference between pretreatment oligo-metastasis and radical prostatectomy recurrent oligo-metastasis. NS, No statistical difference. *P < 0.05. **(A)** SUVmax, **(B)** ISUP, **(C)** Gleason Score.

**Figure 4:**
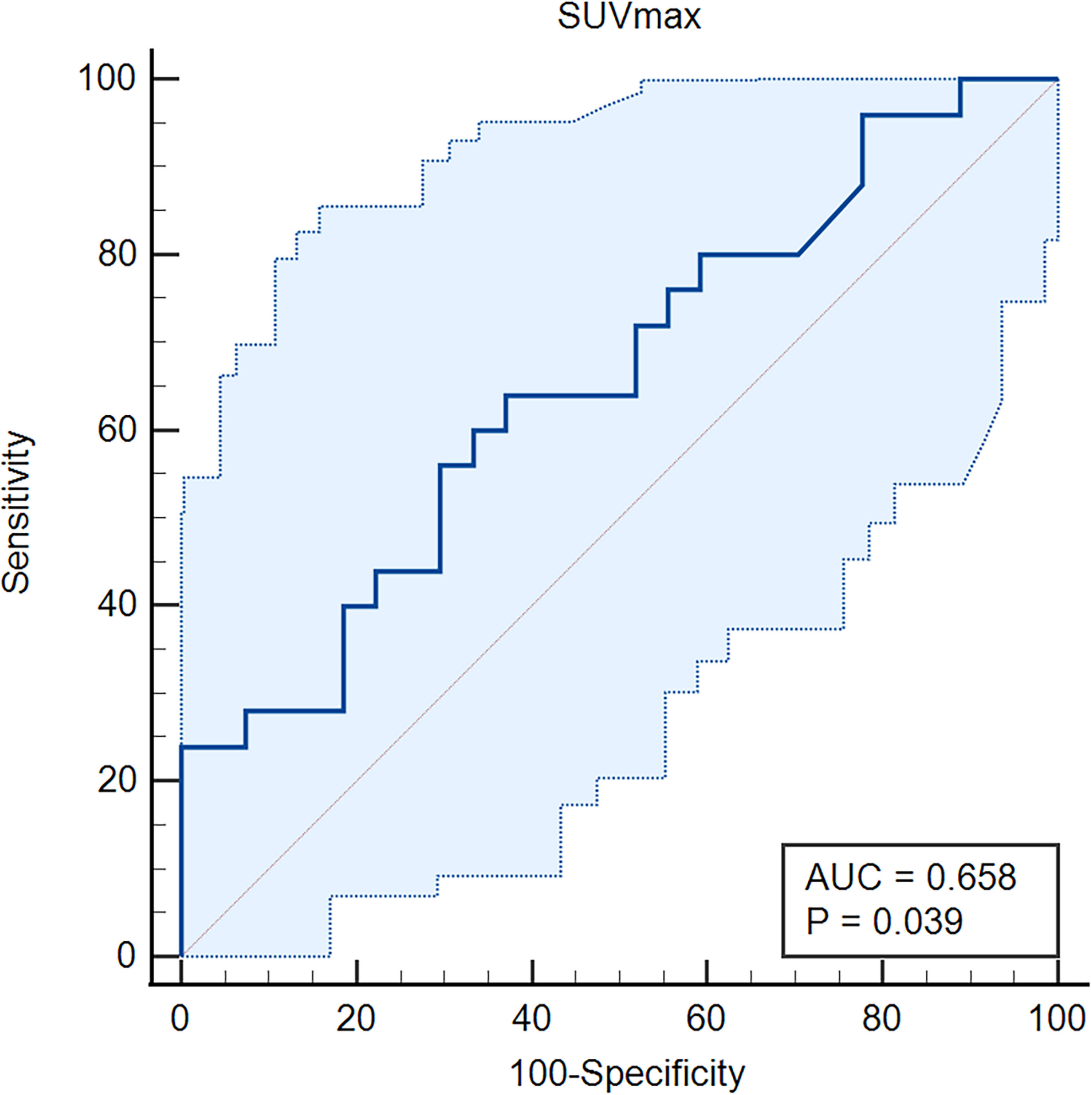
Receiver operating characteristic (ROC) curve of the SUVmax for oligo-metastasis from poly-metastasis discrimination. AUC, Area Under Curve.

Subsequently, we further compared independent predictors between primary oligo-metastasis patients before treatment and after radical prostatectomy without other local or systemic therapy. The characteristics, pathological stage and PSA level of these 69 patients were depicted in **Table 3**. The SUVmax in radical prostatectomy oligo-metastases lesions were significantly higher than in the pretreatment groups (F = 9.993, P = 0.036). There was no statistical difference for the pathological indexes in different oligo-metastases statuses (P = 0.183, P = 0.117, respectively) (**Figure 3**). Referring to the results of the above two cohorts of PCa patients, we use SUVmax to establish the best di-agnostic cut-off value (28.22) for distinguishing oligo-metastasis from poly-metastasis with a sensitivity of 64.00% and specificity of 62.96%, and AUC = 0.658 (95% CI: 0.513 to 0.784, P = 0.039) (**Figure 4**).

**Table 3:** Demographic and clinical characteristics of the different treatment status oligo-metastases.

Characteristic	Value (Overall *n = 69*)
**Age (years)**	
Mean ± SD (range)	69.19 ± 10.23 (42-96)
**PSA (ng/mL)** Mean ± SD (range)	29.08 ± 43.11 (0.003-194.2)
**Oligo-Metastases Status**	
Pretreatment	40 (58.00%)
After radical prostatectomy	29 (42.00%)
**Gleason Score** 6 7 8 9	6 (8.7%) 27 (39.1%) 12 (17.4%) 24 (34.8%)
**ISUP Grade**	
1	6 (8.7%)
2	10 (14.5%)
3	17 (24.6%)
4	12 (17.4%)
5	24 (34.8%)

PCa, prostate cancer; SD, standard deviation; PSA, prostate-specific antigen; ISUP, International Society of Urological Pathology; ISUP Grade 1, Gleason ≤ 6; ISUP Grade 2, Gleason = 3 + 4; ISUP Grade 3, Gleason = 4 + 3; ISUP Grade 4, Gleason = 8; ISUP Grade 5, Gleason>8.

In order to verify whether SUVmax can predict the longitudinal results of oligo-metastases before ADT treatment, we further conducted a follow-up of 22 patients, and the results are shown in **Table S1**. Based on Practical PERCIST 1.0 and Consensus statements on PSMA PET/CT response assessment for disease progression assessment (11–13), 5 patients were considered as progressive disease elevated by metastatic lesions number, SUVmax, the threshold for the standardized uptake value corrected for lean body mass and PSA value. To be specific, all these five oligo-metastasis patients’ SUVmax were higher than 28.22 at baseline (69.02, 46.3, 34.02, 79.15, 63.69, respectively) with different Gleason Score (range: 8-9).

## Discussion

Oligo-metastasis PCa is increasingly recognized as a unique clinical state with therapeutic significance between local disease and widespread metastasis. However, selecting patients that may benefit most from the treatment of oligo-metastasis is an ongoing challenge. Although PSMA overexpression in primary PCa was correlated with advanced tumor malignant status, some clinical guidelines advise against routine PSMA, inducing the possibility of more men presenting with locally advanced or *de novo* oligo-metastasis prostate cancer exists (23). Our prior study has found the ^18^F-PSMA-1007 PET/CT SUVmax has a higher sensitivity and can be an “imaging biomarker” for primary PCa risk stratification and distant metastasis prediction (17). Thus, knowing how best to treat oligo-metastasis patients and make effective predictions in the early stages may become more relevant at a population level.

The PCa metastasis risk prediction included both intra-pelvic and distant metastases, however, the treatment options of different location metastases may be quite different (24, 25). A prior retrospective study has demonstrated that Ga-68 PSMA PET/CT turns out to be a useful tool in determining oligo-metastatic in 50 PCa patients, and the SUVmax value has a positive influence between oligo-metastatic patients and higher metastatic burden (26). Our study extended the comparisons together with non-metastasis patients. SUVmax, PSA value, ISUP and Gleason Score had significant difference among non-metastasis, oligo-metastasis and poly-metastasis status. To be specific, the post-hoc results showed that differences were found between the poly-metastasis sub-groups and the other two groups, but there was no difference between the non-metastasis group and the oligo-metastasis group. The findings in this study are in line with the concept by Weichselbaum and Hellman that oligo-metastasis PCa may represent a unique biologic state with its own natural history compared with extended metastasis (27). Nevertheless, the biological characteristics between non-metastasis and oligo-metastasis may be more similar. Considering to use PSMA PET/CT approach as routine for primary and metastases lesions detection might be more helpful to improve the accuracy of diagnosis and the choice of clinical treatment options.

In addition, our study explored the prediction value of the metastatic degree of primary PCa and SUVmax, ISUP and Gleason Score can be used as independent factors of metastatic burden assessment. Furthermore, for patients with different oligo-metastasis status, the application of the above independent predictor component comparison found that the SUVmax difference between the metastatic lesions between untreated oligo-metastasis and recurrent oligo-metastasis group. Similar to the prior concepts the primary and recurrent oligo-metastatic disease might be represented by the metachronous for distinction biological states (1). This may also help to explain the fact that should be considered when initiating systemic therapies. The metastatic lesions SUVmax were significantly higher in the oligo-recurrent group than in the untreated group. Current data might further support the concept of oligo-metastasis that certain tumors have not fully developed their metastatic potential, the recurrent oligometastatic disease showed a slow natural history and might be more aggressive (2, 27, 28).

Currently, no validated approach to guide optimal therapy for individual oligo-metastasis patients, some studies consider local therapy is sufficient and others though this status is most likely also associated with micro-metastatic disease, therefore, systemic therapy should be considered the optimal treatment and oligo-metastasis PCa might be considered potentially curable with ADT (10, 11, 29). Our study established the SUVmax cut-off 28.22 for discrimination of the oligo and extensive status of metastasis. We further applied this indicator to the follow-up results of ADT patients and found that when the SUVmax of the oligo-metastasis PCa primary lesion was greater than 28.22 at the initial diagnosis, all patients had disease progression after 3 months. Although a cornerstone of treating metastatic prostate cancer, ADT is associated with several deleterious adverse effects and, in some patients, might decrease overall life expectancy (30, 31). Prior studies calculated the proportion of treated metastatic PCa who ever used ADT, with values ranging from 68% to 98% (32–34). Given varying baseline rates of ADT use and varying indications for initiating ADT during follow-up monitoring, quantifying an average effect of directed therapies is difficult (1). This current result might be helpful to select potential oligo-metastasis PCa patients at baseline for controlling the disease with initiated systemic therapy.

Our study was limited by the retrospective data collection and a relatively small sample size. We selected a total of three cohorts for the study, and a more rigorous cohort selection and prospective design may be more helpful to improve the reliability of the results. In addition, this study only initially explored the potential beneficiaries of oligo-metastatic PCa with ADT treatment, and the study on radical prostatectomy and other treatment options still needs to be further expanded.

In summary, the present study found that SUVmax was an independent predictor for both PCa metastasis degree and oligo-metastasis status distinction. For untreated patients with newly diagnosed oligo-metastasis PCa, when the SUVmax of the prostatic carcinoma focus is greater than 28.22, the patient may still have disease progression under ADT treatment. This may provide a reference for the selection of treatment options for baseline ADT.

## Contribution to the field

We searched PubMed for the most relevant research articles on PET/CT oligo-metastasis prostate cancer discrimination using the following terms “oligo-metastasis” and “prostate cancer” and/or “PET/CT”. We found 28 articles on and we compared our results to several previously published association studies.

We identified that only one case report illustrated hormone-sensitive metastatic bone and lymph node flare on ^18^F-PSMA-1007 PET/CT. One study finds Ga-68 PSMA PET/CT turn out to be a useful tool in determining oligometastatic prostate cancer. The remaining studies have focused on the assessment of biochemical recurrence after radical prostatectomy and the post-treatment assessment of radiotherapy. To our knowledge, this is the first study to give a comprehensive picture of the ^18^F-PSMA-1007 PET/CT validation for primary prostate cancer metastasis degree and oligo-metastasis status discrimination.

Our study found that the ^18^F-PSMA-1007 PET/CT SUVmax was an independent predictor for both prostate cancer metastasis degree and oligo-metastasis status distinction. The androgen deprivation therapy treated oligo-metastasis patient may still have disease progression when the primary prostatic carcinoma focus SUVmax is greater than 28.22. This may provide a reference for the selection of treatment options for baseline androgen deprivation therapy.

## Data availability statement

The original contributions presented in the study are included in the article/**Supplementary Material**. Further inquiries can be directed to the corresponding author.

## Ethics statement

The studies involving human participants were reviewed and approved by The study has been approved by the institutional review board (No. 2019LSYZD-J1-H) of the First Affiliated Hospital of Xi’an Jiaotong University. The patients/participants provided their written informed consent to participate in this study.

## Author contributions

ZW draft the manuscript, contributed to the conception and design, analysis and interpretation of data. AZ and YXL contributed to the analysis and interpretation of data. JG, WD and YL contributed to acquisition of data. XD contributed to revised of the manuscript critically for important intellectual content. All authors contributed to the article and approved the submitted version.

## Funding

This research was supported by the National Natural Science Foundation of China (Nos. 82001772), the Natural Science Foundation of Shaanxi Province, China (2021SF-062, 2020JZ-38), and the New Medical and Technology of the First Affiliated Hospital of Xi’an Jiaotong University (XJYFY-2019J1).

## Conflict of interest

The authors declare that the research was conducted in the absence of any commercial or financial relationships that could be construed as a potential conflict of interest.

## Publisher’s note

All claims expressed in this article are solely those of the authors and do not necessarily represent those of their affiliated organizations, or those of the publisher, the editors and the reviewers. Any product that may be evaluated in this article, or claim that may be made by its manufacturer, is not guaranteed or endorsed by the publisher.
