# Supplementary material for: The 18F-PSMA-1007 PET/CT performance on metastasis status and therapy assessment in oligo-metastasis prostate cancer
Source: Front Oncol. 2022 Aug 26;12:935979. doi: 10.3389/fonc.2022.935979 (PMC9458929; doi:10.3389/fonc.2022.935979)
Supplement: Supplementary file 1 [file Table_1.docx]

Supplementary Material

**Table S1** Androgen deprivation therapy response assessment

|  | **Baseline SUVmax before ADT** | **Gleason/ISUP Grade** | **Progressive disease** | **Interval ranged (Month)** |
| --- | --- | --- | --- | --- |
| Patient 1 | 6.16 | 7/2 | - | 14 |
| Patient 2 | 69..02 | 8/4 | **+** | 15 |
| Patient 3 | 10.61 | 9/5 | - | 15 |
| Patient 4 | 8.47 | 9/5 | - | 8 |
| Patient 5 | 13.23 | 9/5 | - | 5 |
| Patient 6 | 7.73 | 9/5 | - | 3 |
| Patient 7 | 46.3 | 8/4 | **+** | 3 |
| Patient 8 | 8.36 | 9/5 | - | 13 |
| Patient 9 | 15.77 | 8/4 | - | 15 |
| Patient 10 | 12.69 | 10/5 | - | 4 |
| Patient 11 | 34.02 | 8/4 | **+** | 5 |
| Patient 12 | 14.58 | 8/4 | - | 6 |
| Patient 13 | 11.18 | 9/5 | - | 6 |
| Patient 14 | 17.74 | 7/3 | - | 5 |
| Patient 15 | 12.40 | 9/5 | - | 3 |
| Patient 16 | 25.64 | 8/4 | - | 15 |
| Patient 17 | 79.15 | 9/5 | **+** | 8 |
| Patient 18 | 63.69 | 9/5 | **+** | 5 |
| Patient 19 | 23.41 | 6/1 | - | 13 |
| Patient 20 | 18.13 | 7/3 | - | 11 |
| Patient 21 | 21.75 | 10/5 | - | 3 |
| Patient 22 | 17.749 | 7/2 | - | 7 |

Note: ADT: Androgen deprivation therapy. ISUP: International Society of Urological Pathology. ISUP Grade 1: Gleason ≤ 6, ISUP Grade 2: Gleason = 3+4, ISUP Grade 3: Gleason = 4+3, ISUP Grade 4: Gleason = 8, ISUP Grade 5: Gleason＞8. -: negative, +: positive.
